# Sex Differences and Extracorporeal Shockwave Therapy Outcomes in Runners with Achilles or Hamstring Tendinopathy

**DOI:** 10.3390/jcm13237360

**Published:** 2024-12-03

**Authors:** Nicole B. Katz, Sydney C. Karnovsky, David M. Robinson, Stephanie E. DeLuca, Phillip H. Yun, Ellen Casey, Meagan M. Wasfy, Adam S. Tenforde

**Affiliations:** 1 Department of Physical Medicine and Rehabilitation, Harvard Medical School, Boston, MA 02115, USA; nkatz@mgh.harvard.edu (N.B.K.); drobinson22@mgb.org (D.M.R.); sdeluca3@mgb.org (S.E.D.); 2 Spaulding Rehabilitation Hospital, Boston, MA 02129, USA; 3 Department of Orthopeadic Surgery and Rehabilitation Medicine, University of Chicago, Chicago, IL 60637, USA; phillip.yun@bsd.uchicago.edu; 4 Department of Physiatry, Hospital for Special Surgery, New York, NY 10021, USA; caseye@hss.edu; 5 Cardiology Division, Massachusetts General Hospital, Boston, MA 02114, USA; mwasfy@mgb.org

**Keywords:** shockwave, tendinopathy, female athletes

## Abstract

**Background/Objectives**: Achilles and hamstring tendinopathies are common injuries in runners, and extracorporeal shockwave therapy (ESWT) may be an effective treatment. Runners are at risk for lower extremity tendinopathies and the Male and Female Athlete Triad (Triad). The purpose of this study is to evaluate the association of sex, exposure to hormonal contraceptives, menopause, and Triad-related risk factors with ESWT outcomes in the treatment of Achilles and hamstring tendinopathy. **Methods**: This is a retrospective cohort study of runners with either Achilles or hamstring tendinopathy who received radial or combined radial and focused ESWT. Tendon function was measured using Victorian Institute of Sport Assessment (VISA) questionnaires completed before and following treatment. Treatment success was defined by a change in VISA score that met the minimal clinically important difference (MCID). **Results**: There were 88 runners (54.5% female, 45.5% male) with Achilles (52.3%) or hamstring (47.7%) tendinopathy. No measurable difference was found in the proportion of females and males overall that achieved the MCID (57.1% and 72.5%, *p* = 0.17). Similar rates of females and males met MCID for Achilles (77.8% and 75.0%, *p* = 0.83) and hamstring tendinopathy (46.7% and 66.7%, *p* = 0.24). However, females with Achilles or hamstring tendinopathy who used oral contraceptive pills (OCPs) were less likely to meet the MCID compared to females not on OCPs (*p* = 0.031); this finding was present in a subgroup analysis of runners with only Achilles tendinopathy (*p* = 0.025). No associations were found between achieving the MCID and Triad risk factors, including body mass index, energy availability, weight-related behaviors, bone health, or menstrual function (all *p* > 0.05). **Conclusions**: Female and male runners reported similar success rates for ESWT, and Triad risk factors were not found to impact outcomes. However, females who used oral contraceptive pills were less likely to achieve the MCID from ESWT.

## 1. Introduction

Running places strong forces on the body that put individuals at an increased risk of injury. In particular, runners are at high risk for lower extremity injuries, with incidence rates ranging from 19.4% while training for a single marathon to 79.3% over a 6-month period [1,2,3]. Knees are the most common site of injury, followed by the lower leg (Achilles tendon, shin, calf, and heel) and then the upper leg (hamstring, thigh, and quadriceps) [1]. While the incidence of hamstring tendinopathy has not been well documented in this population, Achilles tendinopathy has been reported at lifetime rates of 31% in elite short-distance runners and 42% in elite middle- and long-distance runners before the age of 45 [4]. Given the rates of these injuries, efforts have been made to develop treatment protocols for lower extremity tendinopathies that minimize the time away from participation and support a quicker return to running.

The use of extracorporeal shockwave therapy (ESWT) for athletes with musculoskeletal pathologies has been increasing along with the growing literature highlighting its efficacy and unique properties. ESWT applies energy in the form of shockwaves to the area of pathology. ESWT may be applied to bone as well as soft tissues, including tendons, fascia, and muscles [5]. The energy applied may differ by mode of delivery—radial ESWT or focused ESWT. Radial ESWT provides lower peak pressure waves from a pneumatic/ballistic device that acts on more superficial structures, whereas focused EWST delivers higher amplitude waves produced by an electromagnetic, hydraulic, or piezoelectric device at greater depths compared to radial ESWT [5,6,7]. These therapies likely elicit various healing mechanisms, including the destruction of calcifications, angiogenesis in the inflammatory phase of tendon repair, activation of cell signaling pathways through the release of adenosine triphosphate, and anti-inflammatory and analgesic effects through the release of nitric oxide [5,8]. ESWT has been suggested to be an effective treatment for runners with lower extremity tendinopathies and provides the benefit of not requiring time away from sport during treatment [5,9,10].

Additional factors may influence the development of tendinopathy and response to ESWT in runners. Sex hormones may play an important role in the pathophysiology of musculoskeletal injuries, including tendinopathies. Estrogen, in particular, has been found to impact collagen synthesis and turnover as well as the inflammatory pathway crucial to tendon healing [11,12,13]. These discoveries may be clinically relevant to females experiencing varying concentrations of estrogen during the menstrual cycle, pregnancy, or menopause or by exposure to exogenous estrogen in the form of hormonal contraceptives or hormone replacement therapy. There is some evidence suggesting that oral contraceptive pills (OCPs) may increase the risk of tendinopathy [11]. Sex hormones may also be influenced by the nutrition status of athletes and affect female and male runners differently. The Female Athlete Triad (Triad) describes the medical syndrome of three interrelated conditions: (1) low energy availability with or without disordered eating, (2) menstrual dysfunction, and (3) low bone mineral density (BMD) [14]. A similar condition has been defined in male athletes (Male Athlete Triad), with the difference being that reproductive endocrine dysfunction is defined as functional hypothalamic hypogonadism instead of menstrual dysfunction [15]. The Triad is more common in endurance and weight-class athletes as well as those participating in sports that emphasize a lean physique, like running [14,15]. Runners have been found to be at high risk for the Triad, and Triad risk factors may predispose runners to injuries [16,17,18,19,20]. Despite these findings, it is unknown if sex differences, exogenous or endogenous hormones, or Triad-related risk factors influence tendon response to ESWT. A thorough comprehension of the associations between these variables may advance tendinopathy treatment in this population.

The purpose of this study is to evaluate the association of sex, exposure to hormonal contraceptives, menopause, and Triad-related risk factors with ESWT outcomes in the treatment of Achilles and hamstring tendinopathy. Symptoms of tendon severity were described using the Victorian Institute of Sport Assessment-Achilles (VISA-A) and Victorian Institute of Sport Assessment-Hamstring (VISA-H) [21,22], with both assessments establishing minimal clinical important difference (MCID) values for tracking clinically significant improvement in symptoms [22,23,24]. We hypothesized that there would be no differences in achieving the MCID between sexes. We also hypothesized that Triad risk factors and altered estrogen states (hormonal contraceptive use, menstrual dysfunction, and menopause) would not affect ESWT outcomes.

## 2. Materials and Methods

This was a retrospective cohort study. Participants were identified through retrospective chart review of patients who presented to a single physician’s (AST) outpatient sports medicine clinic and received ESWT for Achilles or hamstring tendinopathy between August 2017 and December 2021, with a portion of these outcome data published previously [25,26]. Diagnosis was made through clinical history and physical examination. Imaging, including radiographs, ultrasounds, magnetic resonance imaging, and computed tomography scans, were obtained and reviewed as clinically necessary for diagnosis.

All patients in this clinic were asked to complete intake questionnaires that included questions about their presenting concern, medical history, sports activity, bone health, weight-related behaviors, menstrual history and function, and medication use, including hormonal contraceptives. Patients were also asked to complete a baseline VISA-A or VISA-H prior to beginning ESWT, throughout their treatment, and following completion of their treatment to assess functional outcomes. VISA-A and VISA-H are patient-reported outcome questionnaires validated to assess the clinical severity of Achilles tendinopathy or proximal hamstring tendinopathy, respectively [21,22]. VISA-H was used to assess patients with proximal and distal hamstring tendinopathy in this cohort, given lack of equivalent validated questionnaire for distal hamstring tendinopathy. The VISA-A and VISA-H questionnaires are 8-question surveys that focus on pain and function. For both VISA-A and VISA-H questionnaires, the scores range from 0 to 100, with higher scores representing greater function and less symptom severity [21,22].

Clinical data collected included demographics at time of first visit, clinical characteristics, ESWT protocol specifications, and functional outcomes. Triad-related risk factors collected were defined as outlined in the Triad Cumulative Risk Assessment score developed by The Female Athlete Triad Coalition [14]. This metric stratifies individuals as low, moderate, or high risk for the Triad based on severity of specific clinical characteristics. These risk factors include low energy availability with or without disordered eating or an eating disorder; body mass index (BMI); age of menarche; abnormal uterine bleed (AUB)- infrequent (formerly termed oligomenorrhea) or amenorrhea; prior bone stress injury or stress fracture; and hip, spine, or total body BMD determined using dual-energy X-ray absorptiometry (DXA) [14,27]. Runners were categorized as having amenorrhea or AUB-infrequent if they selected “less than 3” or “4–9”, respectively, when asked, “What is the fewest number of periods you have had over the 12 months” on their medical intake questionnaire. DXA scores were included for patients if completed within 5 years of the initial visit, given the recommendation for DXA scans to be performed every 3–5 years in females at low/moderate risk for low BMD [28,29]. Low BMI was defined as less than 18.5 kg/m^2^. Low energy availability with or without disordered eating or eating disorder was defined as previous or current disordered eating or eating disorder. Weight-related behaviors were defined as worry about weight or effort to lose or gain weight. A repeat check of 10% of the extracted data was conducted to confirm accuracy of data entry.

Participants were included if they (1) self-identified as a runner, (2) received a single diagnosis of Achilles or hamstring tendinopathy, (3) received ESWT for only Achilles or hamstring tendinopathy, (4) completed an initial VISA-A or VISA-H, and (5) completed a final VISA-A or VISA-H. Runners were excluded if they (1) received additional musculoskeletal diagnoses beyond hamstring or Achilles tendinopathy during the duration of their ESWT treatment; (2) received ESWT to additional anatomic locations beyond those targeted for hamstring or Achilles tendinopathy during their ESWT treatment; (3) received prior surgery to their Achilles or hamstring tendon; (4) received an injection of any injectate to the pathologic tendon during ESWT; or (5) were diagnosed with underlying inflammatory, autoimmune, or connective tissue disease.

ESWT was offered as a treatment option to runners with Achilles or hamstring tendinopathy. Since this treatment modality is not covered by commercial insurance in the United States of America, participants paid a standardized one-time treatment fee. All ESWT was protocoled by a sports medicine physician (AST). Participants received radial ESWT (Storz Extracorporeal Pulse Activation Technology (EPAT^®^) device [Storz Medical, Tägerwilen, Switzerland]) or combined radial and focused (the Storz Duolith device [Storz Medical, Tägerwilen, Switzerland]) ESWT based on clinical course and best practices [6]. Radial ESWT involved a minimum of 3000 shocks delivered at 15 Hz with pressure ranging from 1.8 Bar to 5.0 Bar. Focused ESWT involved a minimum of 1000 shocks delivered with energy flux density ranging from 0.07 to 0.5 mJ/mm^2^. ESWT was provided to the pathologic tendon (Achilles or hamstring tendon), and treatment was focused at the anatomic site of increased discomfort (clinical focusing). Topical or systemic anesthetics were not used during treatments, and participants were counseled to avoid use of nonsteroidal anti-inflammatory drugs (NSAIDs) during the duration of the ESWT treatments to avoid interruption of the inflammatory cascade stimulated by ESWT and suggested to be involved in the healing response [8,30]. Participants typically received 3–4 weekly sessions with a 3-month follow up; additional sessions were added based on clinical response and best practices as described previously with no additional treatment fee required [6,31]. Runners were counseled to participate in physical therapy and continue running, if desired, throughout ESWT treatment if pain was consistently below 5 out of 10 on the visual analog scale (VAS).

The VISA-A and VISA-H questionnaires were used to assess the primary outcome of association between functional improvement after ESWT and sex. The minimal clinically important difference (MCID), the smallest change in VISA scores (final VISA score minus initial VISA score) expected to be meaningful to a patient, is 12 for insertional Achilles tendinopathy, 7 for non-insertional Achilles tendinopathy, and 22 for hamstring tendinopathy [22,23,24]. Achieving MCID was used as an indicator of meaningful, functional improvement. Secondary outcomes included assessing the associations between Triad-related risk factors, exposure to hormonal contraceptives, menopause, and meeting MICD.

Statistical analyses included means and standard deviations or medians and interquartile ranges for continuous data and descriptive statistics for categorical data. Statistical significance was set at α = 0.05, and significance was determined using unpaired 2-tailed Student’s *t*-tests, Chi-square tests, Fisher’s exact tests, and Mann–Whitney U tests. For all comparisons of continuous data in which sample sizes were less than 25 in either group, we performed sensitivity analyses using Mann–Whitney U tests to ensure results were robust. Subgroup analyses were performed to evaluate primary and secondary outcomes within each tendinopathy group (Achilles or hamstring tendinopathy). We conducted a post hoc power analysis to detect the difference in proportions achieving the MCID between sexes observed in this study using the sample sizes of this study. As Triad-related risk factors were not available for all runners, the percentages of runners with each Triad related risk factor were calculated based off of the sample size with data available. Age of 52 was used as a cutoff to approximate menopausal status in female runners because it is the average age for menopause in females in the United States of America to support a larger analysis, given the limited sample size of females that reported menopause status [32].

The term “sex” was defined as biology of the individual as opposed to “gender”, which was defined by how each participant (he/she/they) identified.

The institutional review board at MassGeneralBrigham approved this study (protocol 2023P000080), and informed consent was waived for this study due to determination of this work being a quality improvement initiative.

## 3. Results

### 3.1. Participants

#### 3.1.1. Demographics

There were 88 total runners included in this study. Of the 88 runners, over half (n = 48, 54.5%) were female, and the remainder (n = 40, 45.5%) were male, with an average age of 39.4 ± 12.3 and 40.1 ± 11.6 years, respectively (*p* = 0.78, Table 1). Similar number of runners had Achilles tendinopathy (n = 46, 52.3%) and hamstring tendinopathy (n = 42, 47.7%). The Achilles tendinopathy cohort was composed of 18 (39.1%) females and 28 (60.9%) males with similar ages (39.9 ± 11.4 and 40.0 ± 11.0 years, respectively; *p* = 0.97). The hamstring tendinopathy cohort was composed of 30 (71.4%) females and 12 (28.6%) males with similar ages (39.1 ± 13.0 and 40.3 ± 13.4 years, respectively; *p* = 0.79, Table 1).

#### 3.1.2. Tendinopathy Cohort Clinical Characteristics

Female runners with Achilles tendinopathy had a similar median duration of symptoms compared to male runners prior to beginning ESWT (*p* = 0.51, Table 1). This finding was also true for runners with hamstring tendinopathy (*p* = 0.34, Table 1). In both tendinopathy cohorts, there were no differences in total shockwave sessions received between sexes (all *p* > 0.05, Table 1). There were also no significant differences between sexes for initial, baseline, or change in VISA scores for runners with insertional Achilles tendinopathy, non-insertional Achilles tendinopathy, or hamstring tendinopathy (all *p* > 0.05, Table 1).

### 3.2. Cumulative Shockwave Outcomes for Runners with Achilles or Hamstring Tendinopathy

#### 3.2.1. Sex Differences

There was no significant difference between female and male runners with Achilles or hamstring tendinopathy who achieved the MCID. Overall, 28 (57.1%) female runners and 29 (72.5%) male runners achieved the MCID (*p* = 0.17, Table 1).

#### 3.2.2. Triad Related Risk Factors 

Triad-related risk factors were compared for female and male runners who met the MCID and did not meet the MCID, and no associations were found (Table 2). Specifically, for females and males with Achilles or hamstring tendinopathy, there were no significant differences between the Met MCID and Unmet MCID groups for BMI, low BMI, prior bone stress injury, low energy availability, weight-related behaviors, or low BMD (all *p* > 0.05, Table 2). Additionally, there was no significant difference among females between groups for delayed menarche or AUB- infrequent/amenorrhea (all *p* > 0.05, Table 2).

#### 3.2.3. Exposure to Hormonal Contraceptives and Menopause

When comparing female runners with Achilles or hamstring tendinopathy who did and did not achieve the MCID, a significant difference was found in the number taking OCPs. One (3.7%) female runner in the group who met the MCID was taking OCPs, compared to five females (27.8%) in the group who did not meet the MCID and were taking OCPs at the time of ESWT (*p* = 0.031, Table 2), suggesting female runners who used OCPs were significantly less likely to meet the MCID. There was a trend of OCP users being younger (32.5 ± 6.7) than non-users (42.6 ± 13.4), though not significantly different (*p* = 0.09, Table 3). OCP users were also found to be significantly more likely to have had delayed menarche (*p* = 0.034). No further differences in clinical characteristics or Triad risk factors were present between OCP users and non-users (all *p* > 0.05, Table 3).

There were no associations found between achieving the MCID or not and the use of an IUD/implant (8 [29.6%] and 2 [11.1%], *p* = 0.27) or oral hormone replacement (0 [0.0%] and 1 [5.6%], *p* = 0.40, Table 2). Moreover, no significant differences were found between the group that met the MCID and that did not meet the MCID in regard to menopause status with extracted data (24 [85.7%] and 13 [76.5%], *p* = 0.43) or when approximated with an age of less than 52 years, representing pre-menopausal status (24 [85.7%] and 14 [70.0%], *p* = 0.19, Table 2).

### 3.3. Shockwave Outcomes for Runners with Achilles Tendinopathy

#### 3.3.1. Sex Differences

There was no significant difference between female and male runners with insertional or non-insertional Achilles tendinopathy who achieved the MCID. Within the Achilles tendinopathy cohort, 14 (77.8%) female and 21 (75.0%) male runners achieved the MCID (*p* = 0.83, Table 1).

#### 3.3.2. Triad Related Risk Factors

Triad-related risk factors were compared between female and male runners with Achilles tendinopathy who achieved the MCID and did not achieve the MCID, and no associations were found (Table 4). In this subgroup analysis, for females with Achilles tendinopathy, there were no significant differences between the Met MCID and Unmet MCID groups for BMI, prior bone stress injury, weight-related behaviors, low BMD, delayed menarche, or AUB- infrequent/amenorrhea (all *p* > 0.05, Table 4). Within this cohort, no runners had low BMI or energy availability (Table 4).

For males with Achilles tendinopathy, there were no significant differences between the Met MCID and Unmet MCID groups for BMI, prior bone stress injury, or weight-related behaviors (all *p* > 0.05, Table 4). In this cohort, no males had low BMI or energy availability. Out of both groups, only one male in the Met MCID group had a DXA; this participant met the criteria for low BMD (Table 4).

#### 3.3.3. Exposure to Hormonal Contraceptives and Menopause

Female runners with Achilles tendinopathy who took OCPs were significantly less likely to achieve the MCID. No female runners that met the MCID compared to 2 (66.7%) females that did not meet the MCID were taking OCPs at the time of ESWT (*p* = 0.025, Table 4). There was no difference between groups (Met MCID and Unmet MCID) in runners who used IUDs/implants (5 [38.5%] and 0 [0.0%], *p* = 0.51). No runners in this cohort were taking oral hormone replacement. There were also no associations found between the group that met the MCID and the group that did not meet the MCID in regard to menopause with extracted data (12 [85.7%] and 3 [100.0%], *p* = 1.0) or when approximated with age less than 52 years, representing pre-menopausal status (12 [85.7%] and 3 [75.0%], *p* = 1.0, Table 4).

### 3.4. Shockwave Outcomes for Runners with Hamstring Tendinopathy

#### 3.4.1. Sex Differences

There was no significant difference between female and male runners with hamstring tendinopathy who achieved the MCID. Within the hamstring tendinopathy cohort, 14 (46.7%) female runners and 8 (66.7%) male runners achieved the MCID (*p* = 0.24, Table 1).

#### 3.4.2. Triad Related Risk Factors

Triad-related risk factors were compared between female and male runners with hamstring tendinopathy who achieved the MCID and did not achieve the MCID, and no associations were found (Table 4). For females with hamstring tendinopathy, there were no significant differences between the Met MCID and Unmet MCID groups for BMI, low BMI, prior bone stress injury, low energy availability, weight-related behaviors, low BMD, delayed menarche, or AUB-infrequent/amenorrhea (all *p* > 0.05, Table 4).

For males with hamstring tendinopathy, there were no significant differences between the Met MCID and Unmet MCID groups for BMI, prior bone stress injury, or weight-related behaviors (all *p* > 0.05, Table 4). In this cohort, like in the Achilles tendinopathy cohort, no males had low BMI or met criteria for low energy availability. Additionally, no males had DXAs to assess BMD (Table 4).

#### 3.4.3. Exposure to Hormonal Contraceptives and Menopause

Similar to the Achilles tendinopathy cohort, the association of exogenous hormone use and menopause status was assessed in female runners with hamstring tendinopathy. However, in this cohort, there were no differences between groups (Met MCID and Unmet MCID) in runners who used OCPs, oral hormone replacement, or IUDs/implants (all *p* > 0.05, Table 4). While more females who did not meet the MCID were OCP users (3 [20.0%]) than those who did meet the MCID (1 [7.1%), no statistical trend or significance was present (*p* = 0.60). There were also no associations found between the group that met the MCID and the group that did not meet the MCID in regard to menopause with extracted data or when approximated with an age less than 52 years, representing pre-menopausal status (all *p* > 0.05, Table 4).

## 4. Discussion

The purpose of this study is to evaluate the association of sex, exposure to hormonal contraceptives, menopause, and Triad-related risk factors with ESWT outcomes in the treatment of runners with Achilles and hamstring tendinopathy. As hypothesized, no differences were found in achieving the MCID between sexes. Additionally, no associations were found between Triad-related risk factors and EWST outcomes. However, female runners who used OCPs were significantly less likely to achieve the MCID. These findings highlight the importance of understanding the effect of variables differentiating female from male athletes in efforts to optimize care.

This work strengthens the existing literature supporting the use of ESWT for Achilles and hamstring tendinopathies [9,10,33,34,35]. Our findings highlight this therapy as an effective treatment option for adult male and female runners with no sex- or age-based differences in the outcomes. Notably, a post hoc power analysis found a power of 31.9% to detect the difference in proportions achieving the MCID between sexes observed in this study with our sample sizes, suggesting that this study may have been underpowered to detect statistically significant differences. Nonetheless, this study supports clinicians treating runners with Achilles or hamstring tendinopathy with ESWT who have not responded adequately to standard care. This advanced treatment option may be particularly helpful for in-season athletes, given that it is non-invasive and does not require a pause in training [5].

Additionally, we found that Triad-related risk factors (BMI, BMD, menstrual function, energy availability, weight-related behaviors, and prior bone stress injuries) may not impact ESWT outcomes in runners. This is of particular importance in this population that has both a high prevalence of lower extremity tendinopathies as well as Triad and Triad-related risk factors [4,16,17,18,19,20,36,37]. This study suggests that runners with Achilles or hamstring tendinopathy and Triad-related risk factors compared to runners without Triad risk factors benefited from ESWT similarly. The relationships between Triad risk factors, tendon healing, and ESWT outcomes have not been well evaluated, and we hope this work encourages further investigation.

In this study, OCP users were found to be significantly less likely to achieve the MCID compared to non-users. This finding was present in female runners when those with Achilles or hamstring tendinopathy were assessed together and in the subgroup analysis of female runners with only Achilles tendinopathy. The subgroup analysis of female runners with only hamstring tendinopathy that used OCPs showed that they were not significantly less likely to achieve the MCID, though a greater percentage of those who did not meet the MCID were OCP users compared to those who did meet the MCID. Combined estrogen and progesterone OCPs are the most prevalent type of OCPs and are used by more than 100 million women worldwide [38]. In the United States of America, 14.0% of females ages 15 to 49 use OCPs [39]. Through suppressed feedback loops, regular OCP use minimizes hormone fluctuations and may alter (lower or raise) levels of plasma estrogen throughout the menstrual cycle [40,41]. While OCP use may lower levels of plasma estrogen in the general population, the exogenous hormone ingestion may increase levels in females with lower baseline levels of estrogen, such as athletes with Triad risk factors, including hypothalamic amenorrhea [42].

OCP use may affect musculoskeletal health by its effect on both sex and non-sex hormones through a variety of pathways. Estrogen, in particular, has been found to impact the musculoskeletal system and injury risk with estrogen receptors present on tendons, ligaments, and skeletal muscle [43]. Prior research on the effect of estrogen on soft tissue injuries and healing has suggested individuals with higher estrogen states have an increased chance of anterior cruciate ligament (ACL) rupture. Moreover, this relationship was particularly strong during the ovulation phase of the menstrual cycle when estrogen levels spike [11]. Higher estrogen levels have also been suggested to lead to a decrease in type I collagen production [11]. Additionally, increased estrogen states may lead to an increase in type III collagen and elastin, thus leading to a decrease in the tensile strength of tendons, given that type III collagen is thinner than type I collagen [12]. However, this relationship is still under investigation with inconsistent findings. In an in vitro murine study, estrogen deficiency led to a decrease in type I collagen and an increase in type III collagen [13]. Notably, estrogen deficiency has also been correlated with a decrease in nitric oxide production immediately following tendon injury, which impairs the initial inflammatory healing phase of the tendon [13]. Compared to estrogen, the understanding of the impact of progesterone on the musculoskeletal system is more limited, with mixed findings regarding the presence of progesterone receptors on tendons [44,45,46]. However, progesterone may indirectly impact tendons through upregulating relaxin receptors, which promote increased collagen degradation and cause decreased tendon stiffness [45,46]. Notably, this effect may not be present in all tendons. Pearson et al. found that serum relaxin levels impact the stiffness of the patellar tendon but not the medial gastrocnemius tendon [46].

The effect of OCPs, specifically on protein and collagen synthesis, has also been evaluated. In a series of studies by Hansen et al. [47,48], age- and fitness-matched subjects taking OCPs were compared to those not taking OCPs before and after a series of exercises [47,48]. This work found lower rates of collagen synthesis in tendons, bones, and muscle connective tissue in OCP users compared to the control group [48]. This result was theorized to be due to OCPs decreasing serum levels of insulin-like Growth Factor-1 (IGF-1), which serves as an anabolic hormone, increasing collagen synthesis [48]. Female athletes using combined estrogen and progesterone OCPs were also found to have greater chronic low-grade inflammation, as measured by high-sensitivity C-reactive protein (CRP), compared to non-OCP users [49]. Similarly, female athletes who were OCP users had greater oxidative stress, as measured by assays for blood hydroperoxides and antioxidant capacity, than non-users [50].

Though the exact effect of OCPs, estrogen, and progesterone on tendons remains incompletely characterized, it is evident that estrogen levels, both endogenous and exogenous, affect tendon healing and regeneration. Our data in this study support this conclusion, as our results show that OCP use may not only alter the properties of tendon healing but also may significantly impair an essential mechanism of healing generated by ESWT. ESWT works in conditions of tendinopathy through a variety of pathways, including controlling inflammation and promoting tendon remodeling [8]. ESWT promotes angiogenesis in the initial inflammatory phase and cellular proliferation through the upregulation of hormones such as IGF-1 and collagen synthesis via the upregulation of transforming growth factor beta 1 (TGF-B1) and interleukin-6 (IL-6). ESWT also decreases the inflammatory response by regulating nitric oxide levels [8]. Runners taking OCPs may have had a dampened reaction to ESWT with an impaired healing response potentially due to OCPs promoting inflammation and oxidative stress, as well as altered systemic estrogen levels affecting IGF-1 production, collagen (type I and type III collagen) synthesis, and nitric oxide levels.

While this study advances our understanding of factors influencing ESWT outcomes, there are limitations to acknowledge. The research methods limit the ability to determine causation and exclude the potential impact of a placebo effect. However, given the known durations of symptoms prior to ESWT, improvement due to the natural history of the pathology was believed to be less likely. Additionally, VISA-H was used to assess both proximal and non-proximal hamstring tendinopathy despite it being validated for only proximal hamstring tendinopathy [22]. In circumstances where VISA questionnaires have not been created/validated for related tendons, surrogate VISA questionnaires have been used, such as using the VISA- Patellar Tendon created for patellar tendinopathy to evaluate quadriceps tendinopathy [31]. The sample size also limits this analysis, and this work should be replicated in a larger cohort. A minority of female runners used OCPs, which further limited the analysis. OCP use was assessed as a binary (user or non-user), and sample size precluded the analysis of the impact of different types of OCPs (quantities of estrogen and the androgenicity of progesterone). Other notable limitations pertain to the assessment of Triad-related risk factors. Patients were asked about their history or current AUB-infrequent/amenorrhea without specifying if contraceptives were used concurrently. Additionally, low energy availability was defined as previous or current disordered eating or eating disorders, and weight-related behaviors were defined as current worry or effort to lose or gain weight. Validated screening questionnaires and/or objective measures may be necessary to more accurately detect low energy availability, disordered eating, and/or eating disorders [51]. Patients were also asked in one survey question if they were trying to gain or lose weight, which prevented the post hoc assessment of those only trying to lose weight.

## 5. Conclusions

This work highlights that ESWT is similarly effective for adult female and male runners with Achilles or hamstring tendinopathy. Moreover, given that the majority of females and males achieved the MCID, ESWT could be considered for runners with refractory tendinopathy. These findings suggest that age, sex, and Triad-related risk factors may not impair ESWT outcomes; however, OCP use in female runners significantly decreased ESWT outcomes. While we cannot prove the mechanism for our observations, the reduced rate of response to ESWT may potentially be explained by OCP use promoting inflammation and oxidative stress and impacting systemic estrogen levels, which interfere with the healing cascades stimulated by ESWT. Treatment of tendinopathy with ESWT may require clinicians to consider patient OCP use when discussing expected results. Further research is warranted to explore the effect of OCP use (overall and of various formulations) on ESWT outcomes and tendon healing. Greater investigation should also be conducted to determine optimal ESWT protocols for individuals on OCPs, as the qualities of their tissues may differ.

## Figures and Tables

**Table 1:** Demographics, clinical characteristics, and extracorporeal shockwave outcomes of runners with Achilles and hamstring tendinopathy (N = 88) ^a^.

	Females	Males	*p*
Overall (N = 88)			
N	48 (54.5)	40 (45.5)	-
Age	39.4 ± 12.3	40.1 ± 11.6	0.78
Achieved MCID	28 (57.1)	29 (72.5)	0.17
Achilles tendinopathy (N = 46)			
N	18 (39.1)	28 (60.9)	-
Age	39.9 ± 11.4	40.0 ± 11.0	0.97
Symptom duration before ESWT (months)	12.5 (2.5, 23.3)	12.0 (6.0, 41.3)	0.51
Total shockwave sessions	6.4 ± 2.9	5.6 ± 1.9	0.27
Baseline VISA-A, insertional	55.3 ± 14.8	55.0 ± 13.2	0.96
Final VISA-A, insertional	72.2 ± 11.7	68.1 ± 12.7	0.53
Change in VISA-A, insertional	16.8 ± 10.9	13.1 ± 9.2	0.46
Baseline VISA-A, non-insertional	56.4 ± 16.9	50.1 ± 16.3	0.32
Final VISA-A, non-insertional	79.2 ± 8.9	73.6 ± 12.3	0.19
Change in VISA-A, non-insertional	22.8 ± 14.4	23.5 ± 17.5	0.91
Achieved MCID	14 (77.8)	21 (75.0)	0.83
Hamstring tendinopathy (N = 42)			
N	30 (71.4)	12 (28.6)	-
Age	39.1 ± 13.0	40.3 ± 13.4	0.79
Symptom duration before ESWT (months)	6.5 (4.3, 16.5)	13.5 (5.3, 26.0)	0.34
Total shockwave sessions	6.4 ± 3.1	5.2 ± 1.1	0.18
Baseline VISA-H	42.9 ± 16.2	45.8 ± 20.5	0.63
Final VISA-H	63.3 ± 21.3	67.8 ± 15.1	0.51
Change in VISA-H	20.4 ± 17.5	22.0 ± 20.1	0.79
Achieved MCID	14 (46.7)	8 (66.7)	0.24

^a^ Data are presented as X ± SD, median (IQR), or No. (%).

**Table 2:** Cumulative runner characteristics for those with Achilles and hamstring tendinopathy who did and did not achieve MCID (N = 88) ^a^.

**Females ^b^**			
	**MCID Met (N = 28)**	**MCID Unmet (N = 20)**	***p***
Age	37.4 ± 12.0	42.2 ± 12.4	0.22
BMI	20.5 ± 2.0	20.9 ± 2.7	0.56
Low BMI (<18.5 kg/m^2^)	5 (17.9)	3 (15.0)	1.0
Prior bone stress injury	12 (42.9)	7 (36.8)	0.68
Low energy availability ^c^	2 (8.0)	2 (11.8)	1.0
Weight-related behaviors ^d^	5 (20.0)	6 (35.3)	0.49
Low BMD (Z- or T-score < −1)	4 (66.7)	3 (60.0)	1.0
Delayed menarche (≥15 yr)	4 (14.8)	3 (17.6)	1.0
Prior or current AUB-infrequent/amenorrhea	16 (69.6)	7 (53.8)	0.74
Current exogenous hormones use			
Oral contraceptive pill	1 (3.7)	5 (27.8)	**0.031**
Intrauterine device/implant	8 (29.6)	2 (11.1)	0.27
Oral hormone replacement	0 (0.0)	1 (5.6)	0.4
Menopause status			
Age < 52 years old	24 (85.7)	14 (70.0)	0.19
Pre-menopause	24 (85.7)	13 (76.5)	0.43
**Males ^b^**			
	**MCID Met (N = 29)**	**MCID Unmet (N = 11)**	***p***
Age	39.9 ± 12.6	40.7 ± 8.9	0.84
BMI	24.3 ± 3.0	24.7 ± 3.9	0.71
Low BMI (<18.5 kg/m^2^)	0 (0.0)	0 (0.0)	1.0
Prior bone stress injury	5 (20.0)	4 (36.4)	0.42
Low energy availability ^c^	0 (0.0)	0 (0.0)	1.0
Weight-related behaviors ^d^	9 (34.8)	3 (60.0%)	1.0
Low BMD (Z- or T-score < −1)	1 (100.0)	N/A	-

^a^ Data are presented as X ± SD or No. (%), significant *p*-values bolded. BMI: body mass index, BMD: bone mineral density, N/A: not available, AUB-infrequent: abnormal uterine bleeding—infrequent (formerly termed oligomenorrhea). ^b^ Sample sizes for variables with missing data include: Unmet MCID females for BSI N = 19, Unmet MCID females for exogenous hormones N = 18, Unmet MCID females for low energy availability/worry about weight/delayed menarche/pre-menopause N = 17, Unmet MCID females for AUB- infrequent/amenorrhea N = 13, Unmet MCID females for low BMD N = 5; Met MCID females for delayed menarche/exogenous hormones N = 27, Met MCID females for AUB- infrequent/amenorrhea N = 26, Met MCID females for low energy availability/worry about weight N = 25, Met MCID females for low BMD N = 6; Unmet MCID males for low BMD N = 10, Unmet MCID males for low energy availability N = 7, Unmet MCID males for worry about weight N = 5, Unmet MCID males for low BMD N = 0; Met MCID males for prior bone stress injury N = 24, Met MCID males for low energy availability/worry about weight N = 18, and Met MCID males for low BMD N = 1. ^c^ Low energy availability was defined as previous or current disordered eating or eating disorder. ^d^ Weight-related behaviors were defined as current worry about weight or effort to lose or gain weight.

**Table 3:** Female runner characteristics for those with Achilles and hamstring tendinopathy who did and did not use oral contraceptive pills during shockwave treatment (N = 33) ^a,b,c^.

	OCP Users (N = 6)	OCP Non-Users (N = 27)	*p*
Age	32.5 ± 6.7	42.6 ± 13.4	0.09
Symptom duration before ESWT (months)	13.5 (3.8, 18.8)	8 (4.5, 14.5)	0.80
Total shockwave sessions	8.0 ± 5.1	6.2 ± 2.7	0.23
Baseline VISA-A, insertional	-	59.3 ± 17.5	-
Final VISA-A, insertional	-	77.5 ± 7.3	-
Change in VISA-A, insertional	-	18.3 ± 11.1	-
Baseline VISA-A, non-insertional	67.5 ± 7.8	47.3 ± 24.2	0.33
Final VISA-A, non-insertional	77.5 ± 7.8	78.0 ± 12.8	0.96
Change in VISA-A, non-insertional	10.0 ± 0.0	30.8 ± 22.2	0.28
Baseline VISA-H	46.5 ± 5.8	40.9 ± 18.6	0.56
Final VISA-H	61.3 ± 17.3	66.2 ± 23.1	0.69
Change in VISA-H	14.8 ± 18.2	25.3 ± 14.2	0.21
BMI	19.6 ± 1.2	20.9 ± 2.6	0.24
Low BMI (<18.5 kg/m^2^)	0 (0.0)	6 (22.2)	0.56
Prior bone stress injury	2 (33.3)	12 (44.4)	1.0
Low energy availability ^d^	1 (20.0)	2 (8.0)	0.43
Weight-related behaviors ^e^	1 (20.0)	8 (32.0)	1.0
Low BMD (Z- or T-score < −1)	N/A	5 (62.5)	-
Delayed menarche (≥15 yr)	3 (60.0)	2 (7.7)	**0.034**
Prior or current AUB-infrequent/amenorrhea	2 (40.0)	11 (47.8)	1.0
Menopause status			
Age < 52 years old	6 (100.0)	17 (63.0)	0.14
Pre-menopause	6 (100.0)	18 (69.2)	0.30

^a^ Data are presented as X ± SD, median (IQR), or No. (%), significant *p*-values bolded. BMI: body mass index, BMD: bone mineral density, OCP: oral contraceptive pill, AUB-infrequent: abnormal uterine bleeding—infrequent (formerly termed oligomenorrhea). ^b^ Insertional Achilles tendinopathy: N = 0 OCP users, N = 4 OCP non-users; non-insertional Achilles tendinopathy: N = 2 OCP users, N = 4 OCP non-users; hamstring tendinopathy: N = 4 OCP users, N = 19 OCP non-users. ^c^ Sample sizes for variables with missing data include: OCP user for low energy availability/worry about weight/delayed menarche/AUB- infrequent/amenorrhea N = 5; OCP non-user for delayed menarche/menopause status N = 26, OCP non-user for low energy availability/worry about weight N = 25, OCP non-user for AUB- infrequent/amenorrhea N = 23, OCP non-user for low BMD N = 8. ^d^ Low energy availability was defined as previous or current disordered eating or eating disorder. ^e^ Weight-related behaviors were defined as current worry about weight or effort to lose or gain weight.

**Table 4:** Subgroup analyses of runner characteristics for those with Achilles and hamstring tendinopathy who did and did not achieve MCID (N = 88) ^a^.

**Females ^b^**						
	**MCID Met, Achilles** **(N = 14)**	**MCID Unmet, Achilles** **(N = 4)**	***p***	**MCID Met, Hamstring** **(N = 14)**	**MCID Unmet, Hamstring** **(N = 16)**	***p***
Age	38.9 ± 12.0	43.3 ± 9.1	0.52	35.9 ± 12.2	41.9 ± 13.3	0.21
BMI	21.7 ± 1.6	20.8 ± 1.9	0.33	19.4 ± 1.8	20.9 ± 2.9	0.09
Low BMI (<18.5 kg/m^2^)	0 (0.0)	0 (0.0)	1.0	5 (35.7)	3 (18.8)	0.42
Prior bone stress injury	5 (35.7)	1 (25.0)	1.0	7 (50.0)	6 (40.0)	0.59
Low energy availability ^c^	0 (0.0)	0 (0.0)	1.0	2 (15.4)	2 (15.4)	1.0
Weight-related behaviors ^d^	2 (15.4)	1 (25.0)	1.0	3 (25.0)	5 (38.5)	0.67
Low BMD (Z- or T-score < −1)	2 (50.0)	0 (0.0)	0.47	2 (100.0)	3 (60.0)	1.0
Delayed menarche (≥15 yr)	1 (7.1)	1 (25.0)	0.41	3 (23.1)	2 (15.4)	1.0
Prior or current AUB-infrequent/amenorrhea	7 (63.6)	0 (0.0)	0.19	9 (75.0)	7 (58.3)	0.67
Current exogenous hormones						
Oral contraceptive pill	0 (0.0)	2 (66.7)	**0.025**	1 (7.1)	3 (20.0)	0.60
Intrauterine device/implant	5 (38.5)	0 (0.0)	0.51	3 (21.4)	2 (13.3)	0.65
Oral hormone replacement	0 (0.0)	0 (0.0)	1.0	0 (0.0)	1 (6.7)	1.0
Menopause status						
Age < 52 years old	12 (85.7)	3 (75.0)	1.0	12 (85.7)	11 (68.8)	0.27
Pre-menopause	12 (85.7)	3 (100.0)	1.0	12 (85.7)	10 (71.4)	0.36
**Males ^b^**						
	**MCID Met, Achilles** **(N = 21)**	**MCID Unmet, Achilles** **(N = 7)**	***p***	**MCID Met, Hamstring** **(N = 8)**	**MCID Unmet, Hamstring** **(N = 4)**	***p***
Age	39.2 ± 11.9	42.6 ± 7.7	0.49	41.6 ± 14.9	37.5 ± 11.1	0.64
BMI	23.8 ± 3.1	24.7 ± 4.9	0.58	25.7 ± 2.1	24.8 ± 1.3	0.45
Low BMI (<18.5 kg/m^2^)	0 (0.0)	0 (0.0)	1.0	0 (0.0)	0 (0.0)	1.0
Prior bone stress injury	2 (11.8)	2 (28.6)	0.55	3 (37.5)	2 (50.0)	1.0
Low energy availability ^c^	0 (0.0)	0 (0.0)	1.0	0 (0.0)	0 (0.0)	1.0
Weight-related behaviors ^d^	5 (45.4)	1 (33.3)	1.0	3 (42.9)	2 (100.0)	0.44
Low BMD (Z-or T-score < −1)	1 (100.0)	N/A	-	N/A	N/A	-

^a^ Data are presented as X ± SD or No. (%), significant *p*-values bolded. BMI: body mass index, BMD: bone mineral density, N/A: not available, AUB-infrequent: abnormal uterine bleeding—infrequent (formerly termed oligomenorrhea). ^b^ Sample sizes for Achilles tendinopathy for variables with missing data include Unmet MCID females for AUB-infrequent/amenorrhea/exogenous hormones/menopause status N = 3; Met MCID females for worry about weight/exogenous hormones N = 13, Met MCID females for low energy availability N = 12, Met MCID females for AUB- infrequent/amenorrhea N = 11, Met MCID females for low BMD N = 4; Unmet MCID males for worry about weight N = 3; Met MCID males for prior bone stress injury N = 17, Met MCID males for low energy availability/worry about weight N = 11, Met MCID males for low BMD N = 1. Sample sizes for hamstring tendinopathy for variables with missing data include: Unmet MCID females for BSI/exogenous hormones N = 15, Unmet MCID females for pre-menopause N = 14, Unmet MCID females for low energy availability/worry about weight/delayed menarche N = 13, Unmet MCID females for AUB- infrequent/amenorrhea N = 12, Unmet MCID females for low BMD N = 5; Met MCID females for low energy availability/delayed menarche N = 13, Met MCID females for worry about weight/AUB- infrequent/amenorrhea N = 12, Met MCID females for low BMD N = 2; Unmet MCID males for low energy availability/low BMD N = 3, Unmet MCID males for worry about weight N = 2; Met MCID males for low energy availability/worry about weight N = 7, Met MCID males for low BMD N = 1. ^c^ Low energy availability was defined as previous or current disordered eating or eating disorder. ^d^ Weight-related behaviors were defined as current worry about weight or effort to lose or gain weight.

## Data Availability

The data that support the findings of this study are available upon request from the corresponding author.
